# Cardio-Dance Exercise to Improve Cognition and Mood in Older African Americans: A Propensity-Matched Cohort Study

**DOI:** 10.1093/geroni/igab046.2882

**Published:** 2021-12-17

**Authors:** Bernadette Fausto

**Affiliations:** Rutgers University-Newark, Newark, New Jersey, United States

## Abstract

The current study sought to determine the influence of initial sleep quality and body mass index on the cognitive and mood outcomes of a community-based cardio-dance exercise program. Thirty-two older African Americans who participated in a five-month cardio-dance exercise program were propensity-matched to 32 no-contact controls (ages 60 to 88). Participants completed neuropsychological tests of attention, executive function, and memory and a self-reported depression measure at baseline and post-test. Among exercise participants, we observed significant improvements in depression (ηp2=.12, p=.009) and attention (ηp2=.12, p=.009) relative to controls. Improvements in executive function and attention were most pronounced among exercise participants with poor sleep quality (ηp2=.41, p=.04) and with obesity (ηp2 = .30, p=.001), respectively. This study provides novel evidence that cardio-dance exercise has the potential to improve depression in older African Americans. For those with poor sleep quality or obesity, exercise can also improve some cognitive outcomes.

